# ASQ-3 developmental outcomes at six months in a Bulgarian infant cohort: a cross-cultural comparison with U.S. norms

**DOI:** 10.3389/fped.2026.1836394

**Published:** 2026-07-20

**Authors:** Tatyana Itova

**Affiliations:** Department of Public Health and Health Care, University of Ruse, Ruse, Bulgaria

**Keywords:** ASQ-3, cross-cultural comparison, developmental screening, early child development, infant development, low birth weight, perinatal risk factors

## Abstract

**Background:**

Early developmental screening relies heavily on U.S.-standardized normative benchmarks, raising questions about cross-cultural stability and the relative influence of biological vs. contextual factors during infancy. This study examined Ages and Stages Questionnaire (ASQ-3) outcomes at six months in a Bulgarian infant cohort in comparison with established U.S. norms.

**Methods:**

In this cross-sectional study, 155 infants (six months chronological or corrected age) were assessed using the ASQ-3 during routine pediatric visits. One-sample *t*-tests were used to compare domain scores with U.S. normative means. Effect sizes (Cohen's d), 95% confidence intervals, and *post hoc* power sensitivity analyses were calculated. Secondary analyses evaluated the effects of birth weight, gestational age, and sex.

**Results:**

Cross-cultural comparisons showed statistically significant differences in Gross Motor (*d* = −0.28) and Problem Solving (*d* = 0.30); however, all effect sizes were small (|d| ≤ 0.30), with no moderate or large deviations observed. In contrast, infants with birth weight <2,500 g demonstrated moderate-to-large effects across multiple domains (*d* = −0.63 to −0.88), particularly in Gross Motor and Personal-Social functioning. Differences related to gestational age were not clinically meaningful after correction for age. A small female advantage was observed in Communication (*d* = 0.37).

**Conclusions:**

ASQ-3 outcomes at six months suggest a high degree of cross-cultural similarity in this Southeastern European infant cohort. Variability in developmental outcomes in the present cohort appeared more strongly associated with biological risk factors than with cross-cultural contextual variation during early infancy. These findings support the use of ASQ-3 in the Bulgarian clinical context and highlight the importance of integrating perinatal risk factors into early developmental surveillance.

## 1 Introduction

Early identification of developmental variation during infancy is a core component of preventive pediatric care and neurodevelopmental surveillance. The first year of life is characterized by rapid motor integration, emerging motor–cognitive coupling, and heightened neuroplastic sensitivity, making this period particularly responsive to both biological and environmental influences (1–3).

The Ages and Stages Questionnaires®, Third Edition (ASQ-3), is one of the most widely implemented parent-report developmental screening tools globally (4, 5). It has demonstrated robust psychometric properties and feasibility across diverse healthcare systems (6, 7) and is increasingly incorporated into population-level developmental surveillance frameworks (8). Longitudinal studies indicate that early ASQ-3 performance shows modest predictive associations with later cognitive outcomes (9), while recent reviews confirm its prominence among globally recommended screening instruments (10).

Despite its widespread use, ASQ-3 normative benchmarks are primarily derived from U.S.-based standardization samples (4, 5), raising important questions regarding cross-cultural stability and the transferability of normative values.

Recent international validation studies provide important context. Psychometric evaluation in Uruguay demonstrated preservation of domain structure across populations (11). Cultural adaptation studies in India confirmed acceptable reliability following contextual modifications (12), whereas standardization efforts in Iran identified domain-specific distributional shifts requiring recalibration of cut-off values (13). Similarly, analyses from Guatemala suggest that variability in certain domains, particularly Fine Motor skills, may reflect culturally influenced caregiving practices rather than true developmental differences (14).

In parallel, biological risk factors play a critical role in early neurodevelopment. Low birth weight and prematurity are consistently associated with vulnerability in motor and cognitive domains (1, 2, 15). The use of corrected age in preterm infants remains essential to avoid overestimation of developmental delay (16). Additionally, small but consistent sex differences, particularly in early communication, have been reported across populations (17, 18).

The six-month developmental period represents a particularly informative and clinically sensitive stage for early developmental surveillance. At this age, foundational motor, communicative, and problem-solving functions are rapidly emerging, while neuroplastic responsiveness remains elevated and environmental influences remain comparatively limited relative to later developmental stages (1–3). Previous developmental screening studies further suggest that developmental variation detected during infancy may demonstrate predictive associations with later cognitive and behavioral outcomes, supporting the clinical value of early identification and monitoring (9). From a clinical perspective, six-month assessments are commonly incorporated into routine pediatric preventive care frameworks internationally, facilitating population-based developmental screening, corrected-age evaluation in preterm infants, and timely referral when developmental vulnerability is suspected (6, 8, 10, 16). Accordingly, examination of ASQ-3 performance at six months provides a clinically meaningful framework for evaluating early developmental patterns and cross-cultural comparability during infancy. Early developmental surveillance during infancy is additionally considered critical for timely identification of developmental vulnerability and initiation of early intervention strategies (19). Recent evidence further supports the clinical utility and comparative accuracy of standardized developmental screening instruments during early childhood surveillance (20).

Developmental outcomes are further shaped by socio-environmental factors, including family-level cognitive stimulation, parenting practices, and modifiable exposures such as screen time (21–24). Accordingly, interpretation of developmental screening results requires a biopsychosocial framework integrating biological, cultural, and environmental influences.

Against this background, the present study examined developmental outcomes at six months in a Bulgarian infant cohort using the ASQ-3 and compared domain-specific scores with established U.S. normative benchmarks. In addition to testing statistical differences, effect size magnitude and statistical sensitivity were incorporated to distinguish statistically significant findings from clinically meaningful variation. Secondary analyses explored the influence of birth weight, gestational age, and sex.

By integrating cross-cultural comparison with biologically relevant subgroup analyses, this study aims to clarify whether observed variability reflects true developmental divergence or expected population-level variation. To date, cross-cultural stability of ASQ-3 at six months has not been evaluated in Southeastern European populations. The present study therefore provides novel regional evidence within an effect size–informed comparative framework.

## 2 Materials and methods

### 2.1 Study design and participants

This cross-sectional study was conducted between August and December 2025 in Ruse, Bulgaria. Participants were recruited during routine six-month pediatric health examinations at the University Multiprofile Hospital for Active Treatment “Medica Ruse” Ltd.

A total of 159 infants were initially enrolled. Of these, 155 with complete and valid ASQ-3 questionnaires were included in the final analysis. Four questionnaires were excluded due to incomplete domain responses. No domain-level missing data were present in the final analytic sample.

Infants were eligible if they were aged six months, defined as either chronological age of six months (±2 weeks) or corrected age of six months for infants born late preterm, in accordance with ASQ-3 administration guidelines. All analyses involving preterm infants were based on corrected age to ensure developmental comparability.

Corrected age was calculated based on standard correction to 40 weeks' gestation.

Infants with diagnosed neurological disorders, major congenital anomalies, or severe perinatal complications were excluded.

Gestational age and birth weight were obtained from medical records.

Given the absence of nationally standardized Bulgarian norms, U.S. normative values served as external reference benchmarks, consistent with international cross-cultural validation studies.

The mean gestational age was 38.1 ± 1.2 weeks. Late preterm birth (34–36 weeks) was observed in 8.4% of infants (*n* = 13). The mean birth weight was 3,280 ± 410 g, with 7.7% (*n* = 12) of infants having a birth weight between 2,000 and 2,500 g. These cohort characteristics were broadly comparable to national perinatal indicators reported in Bulgaria. National epidemiological data indicate that preterm birth rates in Bulgaria are approximately 8%–10% of live births, while the proportion of newborns with birth weight below 2,500 g was reported at 9.38% in 2024 according to the National Statistical Institute. Accordingly, the distribution of gestational age and birth weight in the present cohort appears generally consistent with the broader Bulgarian perinatal population, suggesting reasonable comparability with respect to major biological risk indicators (25, 26).

Detailed sex-stratified normative subgroup data from the original U.S. ASQ-3 standardization sample were not publicly available in the technical documentation and therefore could not be incorporated into the present comparative analyses.

### 2.2 Ethical considerations

The study protocol was reviewed and approved by the Ethics Committee of the University Multiprofile Hospital for Active Treatment “Medica Ruse” Ltd., Bulgaria (Approval No. A-471/01.08.2025). The study was conducted in accordance with the ethical standards of the Declaration of Helsinki. Written informed consent was obtained from all parents or legal guardians prior to participation.

### 2.3 Instrument and data collection procedure

Developmental outcomes were assessed using the six-month form of the Ages and Stages Questionnaire, Third Edition (ASQ-3). Following provision of written informed consent, parents completed the questionnaire remotely. The questionnaires were administered in a distance-based format, allowing parents to complete the forms outside of the clinical visit while maintaining linkage to the routine six-month examination.

The ASQ-3 evaluates five developmental domains:

- Communication
- Gross Motor
- Fine Motor
- Problem Solving
- Personal-Social

Each domain consists of six items scored as 0 (“Not yet”), 5 (“Sometimes”), or 10 (“Yes”), yielding a maximum score of 60 per domain.

### 2.4 Statistical analysis

Statistical analyses were performed using IBM SPSS Statistics (Version 19; IBM Corp., Armonk, NY, USA). Prior to inferential testing, normality assumptions were assessed using Shapiro–Wilk tests, along with inspection of skewness and kurtosis indices.

To compare Bulgarian sample means with U.S. normative values, one-sample *t*-tests were conducted for each developmental domain. Statistical significance was set at *α* = 0.05 (two-tailed). Standardized mean differences (Cohen's d) were calculated using U.S. normative standard deviations as the reference denominator to quantify the magnitude of deviation. Effect sizes were interpreted according to conventional thresholds (0.20 = small, 0.50 = moderate, 0.80 = large) (27). Cohen's d was selected to maintain consistency across the primary cross-cultural comparisons and secondary subgroup analyses, facilitating direct interpretability of effect size magnitude across domains. Although Hedges' g may provide a small-sample correction in subgroup analyses with unequal group sizes, the expected correction factor for the present sample structure was minimal and was not considered likely to materially alter interpretation of effect magnitude.

Independent samples *t*-tests were used to examine subgroup differences according to birth weight, gestational age, and sex, with variance assumptions evaluated using Levene's test. Ninety-five percent confidence intervals were calculated for both mean differences and effect size estimates.

Given the exploratory nature of subgroup analyses, adjustments for multiple comparisons were not applied; results are therefore interpreted in conjunction with effect size magnitude rather than *p*-values alone. As the primary objective was mean-level normative comparison rather than predictive modeling, multivariable analyses were not performed.

A *post hoc* power sensitivity analysis was conducted to estimate the minimum detectable effect size at 80% power given the final sample size (*N* = 155).

## 3 Results

### 3.1 Sample characteristics

The final analytical sample consisted of 155 infants assessed at six months of age. Late preterm infants (<37 weeks’ gestation) accounted for 8.4% of the cohort, while 7.7% had a birth weight below 2,500 g. Female infants comprised 36% of the sample. Recruitment was based on consecutive participation during routine pediatric examinations rather than sex-stratified sampling; therefore, the observed sex imbalance was considered most likely attributable to random variation during the recruitment period. Corrected age was applied for preterm infants in accordance with ASQ-3 guidelines (Table 1).

**Table 1:** Sample characteristics of the Bulgarian infant cohort (*N* = 155).

Characteristic	Value
Total sample size	155
Recruitment period	August–December 2025
Age at assessment	6 months (± 2 weeks; chronological or corrected)
Female infants	36% (*n* = 56)
Male infants	64% (*n* = 99)
Gestational age (weeks)	38.1 ± 1.2
Gestational age > 34 & <37 weeks	8.4% (*n* = 13)
Birth weight (g)	3,280 ± 410
Birth weight > 2,000 & <2,500 g	7.7% (*n* = 12)
Exclusion criteria	Neurological disorders, major congenital anomalies, severe perinatal complications
Ethical approval	Ethics Committee, University Multiprofile Hospital “Medica Ruse” Ltd.

Corrected age was applied for infants born <37 weeks gestational age in accordance with ASQ-3 administration guidelines.

### 3.2 Descriptive developmental outcomes

Shapiro–Wilk tests, together with inspection of skewness and kurtosis indices, indicated no major violations of normality; therefore, parametric analyses were retained.

Mean scores across the five ASQ-3 domains were broadly comparable to U.S. normative benchmarks. Communication and Problem Solving domains showed slightly higher mean values relative to U.S. norms, whereas Gross Motor demonstrated a modest downward shift. Fine Motor and Personal-Social domains exhibited minimal deviation. Overall, descriptive patterns indicated close alignment with reference distributions.

For cross-cultural comparisons, Cohen's d was standardized using U.S. normative standard deviations. For within-sample subgroup analyses, effect sizes were calculated using pooled standard deviations.

### 3.3 Comparison with U.S. normative values

**Figure 1:**
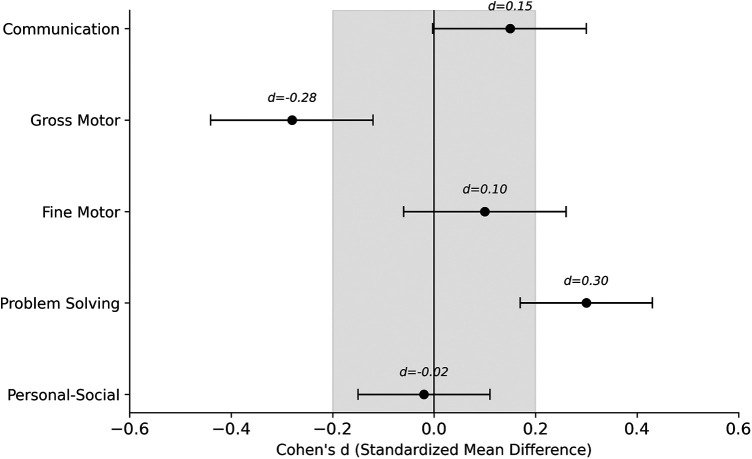
Standardized mean differences (Cohen's d) between the Bulgarian infant cohort and U.S. ASQ-3 normative values across developmental domains at six months. Error bars represent 95% confidence intervals. All observed cross-cultural effect sizes remained within the small range (|d| ≤ 0.30).

One-sample *t*-tests were conducted to evaluate deviations from U.S. normative means (Figure 1).

#### 3.3.1 Communication

The mean Communication score (M = 50.19, SD = 8.33) did not significantly differ from the U.S. normative mean of 48.90, t(154) = 1.93, *p* = 0.055. The mean difference was +1.29 points (95% CI: −0.03 to 2.61), with a small standardized effect size (*d* = 0.15).

#### 3.3.2 Gross motor

Gross Motor scores (M = 42.16, SD = 12.34) were significantly lower than the U.S. normative mean of 45.64, t(154) = −3.51, *p* = 0.001. The mean difference was −3.48 points (95% CI: −5.44 to −1.52), with a small effect size (*d* = −0.28).

#### 3.3.3 Fine motor

No statistically significant difference was observed in the Fine Motor domain (M = 50.12, SD = 11.76) compared to the U.S. normative mean of 48.93, t(154) = 1.26, *p* = 0.209. The mean difference was +1.26 points (95% CI: −0.71 to 3.23), with a small effect size (d = 0.10).

#### 3.3.4 Problem solving

Scores in the Problem Solving domain (M = 53.45, SD = 8.07) were significantly higher than the normative mean of 50.41, t(154) = 4.69, *p* < 0.001. The mean difference was +3.04 points (95% CI: 1.76 to 4.32), with a small effect size (*d* = 0.30).

#### 3.3.5 Personal-Social

No statistically significant difference was observed in the Personal-Social domain (M = 48.10, SD = 9.67) compared to the U.S. normative mean of 48.31, t(154) = −0.27, *p* = 0.784. The mean difference was −0.14 points (95% CI: −1.14 to 0.86), with a negligible effect size (*d* = −0.02).

### 3.4 Summary of cross-cultural comparisons

Two domains (Gross Motor and Problem Solving) reached statistical significance; however, all standardized mean differences were small in magnitude (|d| ≤ 0.30). No moderate or large cross-cultural deviations were detected.

### 3.5 Power sensitivity analysis

A *post hoc* sensitivity analysis indicated that, with a sample size of *N* = 155 and *α* = 0.05 (two-tailed), the study achieved approximately 80% statistical power to detect effect sizes of *d* = 0.23, indicating adequate sensitivity for detecting small-to-moderate deviations.

### 3.6 Secondary analysis by birth weight

Independent samples *t*-tests comparing infants with birth weight < 2,500 g (*n* = 12) and ≥2,500 g (*n* = 143) demonstrated significantly lower developmental scores in the lower birth weight group across multiple domains (Figure 1).

Moderate-to-large effect sizes were observed across several domains, including Communication (*d* = −0.63), Gross Motor (*d* = −0.88), Problem Solving (*d* = −0.72), and Personal-Social functioning (*d* = −0.86). The largest effect was observed in Gross Motor functioning.

In contrast, no meaningful difference was observed in the Fine Motor domain (*d* = −0.07). However, given the relatively small number of infants in the low birth weight subgroup (*n* = 12), these findings should be interpreted with appropriate caution despite the observed effect size magnitude.

### 3.7 Secondary analysis by gestational Age

Comparisons between infants born at <37 weeks' gestation (*n* = 13) and ≥37 weeks (*n* = 142) revealed no statistically significant differences across the Communication, Gross Motor, Problem Solving, or Personal-Social domains (Figure 1)

A non-significant trend was observed in the Fine Motor domain (*p* = 0.078), with a moderate effect size (*d* = 0.52). Given the small size of the preterm subgroup, this finding should be interpreted with caution.

Overall, gestational age status did not meaningfully alter the developmental profile of the cohort at six months.

### 3.8 Secondary analysis by sex

Given the uneven sex distribution within the cohort, subgroup analyses were performed to evaluate potential effects of sex on developmental outcomes. Independent samples *t*-tests revealed a statistically significant difference in the Communication domain, with girls demonstrating higher scores than boys (mean difference = 3.05, 95% CI: 0.08–6.02, *p* = 0.044). The corresponding effect size was small (Cohen's d = 0.37). No statistically significant sex differences were observed in the Gross Motor, Fine Motor, Problem Solving, or Personal-Social domains (Figure 1)

Overall, sex-related variation was limited and did not meaningfully influence the developmental profile of the cohort.

Detailed subgroup comparisons, including degrees of freedom, effect sizes, and variance assumptions, are presented in Table 2.

**Table 2:** Subgroup differences across ASQ-3 developmental domains at six months.

Subgroup	Domain	Subgroup	*n*	Mean ± SD	T (df = 153)	*p*-value	Cohen's d
Birth weight	Communication	<2,500 g	12	45.00 ± 11.48	−2.09	.039	0.53
		≥2,500 g	143	50.27 ± 7.91			
	Gross Motor	<2,500 g	12	33.75 ± 12.45	−2.89	.005	0.88
		≥2,500 g	143	43.93 ± 11.50			
	Fine Motor	<2,500 g	12	49.58 ± 12.52	−0.21	.833	0.07
		≥2,500 g	143	50.35 ± 11.87			
	Problem Solving	<2,500 g	12	47.92 ± 9.16	−2.39	.018	0.66
		≥2,500 g	143	53.97 ± 8.25			
	Personal-Social	<2,500 g	12	41.67 ± 10.94	−2.83	.005	0.82
		≥2,500 g	143	49.51 ± 8.93			
Gestational Age	Communication	<37 weeks	13	51.54 ± 9.66	0.81	.422	0.21
		≥37 weeks	142	49.55 ± 8.27			
	Gross Motor	<37 weeks	13	43.08 ± 10.91	0.04	.966	0.01
		≥37 weeks	142	42.93 ± 12.09			
	Fine Motor	<37 weeks	13	55.77 ± 6.72	1.78	.078	0.52
		≥37 weeks	142	49.63 ± 12.21			
	Problem Solving	<37 weeks	13	55.00 ± 5.77	0.72	.472	0.24
		≥37 weeks	142	53.20 ± 8.76			
	Personal-Social	<37 weeks	13	48.08 ± 7.51	−0.27	.786	0.08
		≥37 weeks	142	48.83 ± 9.61			
Sex	Communication	Female	56	51.45 ± 7.80	2.03	.044	0.36
		Male	99	48.41 ± 8.68			
	Gross Motor	Female	56	44.09 ± 11.27	0.96	.341	0.17
		Male	99	42.03 ± 12.44			
	Fine Motor	Female	56	49.73 ± 12.56	−0.46	.649	0.08
		Male	99	50.71 ± 11.39			
	Problem Solving	Female	56	53.09 ± 9.20	−0.35	.730	0.07
		Male	99	53.62 ± 7.95			
	Personal-Social	Female	56	49.00 ± 10.02	0.26	.792	0.05
		Male	99	48.55 ± 8.92			

Independent samples *t*-tests were conducted, with variance assumptions evaluated using Levene's test. Where appropriate, results from the “equal variances not assumed” condition were reported. Cohen's d was calculated using pooled standard deviations. Effect size magnitude was interpreted according to conventional benchmarks (0.20 = small, 0.50 = moderate, 0.80 = large).

## 4 Discussion

### 4.1 Principal findings

The present study examined ASQ-3 outcomes at six months in a Bulgarian infant cohort relative to U.S. normative benchmarks. Overall, findings suggest a high degree of cross-cultural similarity across domains. Although statistically significant differences were observed in selected domains, standardized effect sizes remained small, and no moderate or large deviations were identified.

This pattern suggests statistical detectability without clinically meaningful divergence, supporting the structural robustness of the ASQ-3 framework beyond its original U.S. standardization sample. While formal measurement invariance testing was beyond the scope of the present design, prior international research has demonstrated structural stability and cross-context applicability of ASQ-3 domains across diverse populations (11, 28, 29).

### 4.2 Cross-cultural interpretation

Although minor domain-level variation was observed, effect sizes remained small and did not indicate structural developmental divergence. International validation studies consistently report preservation of domain structure across diverse populations (11–13). Domain-specific variability, such as that observed in Fine Motor tasks in Guatemala, has been attributed to culturally shaped caregiving practices rather than intrinsic neurodevelopmental differences (14).

The observed pattern of domain-specific differences may also reflect subtle variation in early caregiving environments and parental interaction practices between populations. Cross-cultural parenting frameworks further suggest that caregiving beliefs, developmental expectations, parent–infant interaction styles, and opportunities for early motor exploration may systematically influence the expression and reporting of developmental behaviors across settings while preserving the underlying developmental structure (21–24, 30). In the present cohort, Gross Motor scores were modestly lower relative to U.S. norms, whereas Problem Solving scores were slightly higher. Although the magnitude of these differences was small, variation in early motor experiences, caregiver responsiveness during play, and parent interpretation of questionnaire items have been suggested as potential contributors to minor domain-level variability without indicating structural developmental divergence (12, 14, 30).

The present findings indicate that during early infancy, biologically driven maturational processes may exert a stronger influence on developmental variability than cross-cultural contextual factors. Cultural influences may become more prominent at later developmental stages, when higher-order cognitive and language processes are increasingly shaped by environmental input.

### 4.3 Biological risk factors

In contrast to the limited cross-cultural differences, subgroup analyses revealed more pronounced effects associated with low birth weight. Infants with birth weight < 2,500 g demonstrated moderate-to-large effect sizes across multiple domains, particularly in Gross Motor and Personal-Social functioning. These findings are consistent with extensive evidence linking perinatal vulnerability to early alterations in motor coordination and neurobehavioral organization (1, 2, 15).

Motor domains may be particularly sensitive to early neurodevelopmental perturbations due to their reliance on rapid myelination, corticospinal tract maturation, and sensorimotor integration during early infancy. Similar patterns of motor vulnerability have been reported in preterm populations undergoing developmental screening (31), as well as in infants with growth restriction (32).

No clinically meaningful differences were observed for gestational age following corrected age adjustment. This finding supports established recommendations for age correction in preterm developmental assessment (17) and aligns with recent population-based evidence indicating that corrected age reduces early gestational variability at six months (33, 34).

Given the modest size of biological risk subgroups, these findings should be interpreted cautiously; however, the observed pattern is consistent with existing literature.

### 4.4 Sex differences

A small effect favoring girls in the Communication domain was observed, consistent with evidence of modest female advantage in early language development across populations (17, 18). No clinically meaningful sex differences were identified in other domains. Neurodevelopmental models suggest that such differences may reflect variation in the timing of neural network specialization underlying early communicative processing (35). Overall, the limited magnitude of observed sex-related differences suggests that the uneven sex distribution within the cohort was unlikely to substantially influence the overall developmental profile of the sample.

### 4.5 Statistical versus clinical significance

Although certain domain comparisons reached statistical significance, all cross-cultural effect sizes remained within the small range. Given the study's adequate statistical power, the absence of moderate or large effects supports the interpretation that observed differences are unlikely to reflect clinically meaningful divergence.

Distinguishing statistical significance from clinical relevance is particularly important in developmental screening research, where implementation frameworks prioritize classification utility over isolated *p*-values (36).

### 4.6 Clinical and research implications

The findings support the applicability of the ASQ-3 at six months within the Bulgarian clinical context. Minor domain-specific variation does not appear to warrant structural modification of screening cut-offs. The present findings should also be interpreted within the broader international literature on ASQ-3 adaptation and standardization. Previous studies from multiple cultural settings, including Iran, India, Uruguay, and other Middle Eastern settings, have demonstrated that although the core developmental structure of ASQ-3 is generally preserved across populations, domain-level score distributions and optimal screening thresholds may vary according to sociocultural and contextual factors (11–13, 37). These international standardization efforts highlight the importance of evaluating local normative performance while maintaining comparability with the original ASQ-3 framework. In this context, the present study contributes preliminary Bulgarian cross-cultural comparative data at six months of age and supports the broader international evidence suggesting substantial structural stability of ASQ-3 across diverse healthcare and cultural environments. Nevertheless, the present findings should not be interpreted as a substitute for formal population-based standardization procedures. Future large-scale studies establishing Bulgarian normative reference data across developmental age intervals would further strengthen interpretability, optimize cut-off calibration, and improve population-level screening precision. The global feasibility and scalability of ASQ-3 across healthcare systems have been previously demonstrated (29), further supporting its integration into routine developmental surveillance frameworks. International implementation studies further support the applicability of ASQ-based developmental surveillance frameworks across low- and middle-income healthcare settings (38). Recent evidence additionally demonstrates the feasibility of culturally adapted ASQ-3 versions in identifying developmental risk among preterm infants in non-Western healthcare settings, including Middle Eastern populations (37). Studies examining parent and professional acceptability of ASQ-3 within routine surveillance programs further support its integration into standardized developmental review frameworks (39). These findings reinforce the cross-context applicability of structured parent-report screening tools.

Emerging evidence underscores the importance of integrating perinatal risk profiling and environmental context into screening interpretation (21–23). Future multi-center longitudinal research may further clarify developmental trajectories and predictive validity beyond infancy.

### 4.7 Strengths and limitations

This study has several strengths. It provides systematic cross-cultural comparison using standardized effect size metrics, incorporates confidence intervals and sensitivity analysis, and includes biologically relevant subgroup analyses.

Limitations should also be acknowledged. The study was conducted in a single clinical center, which may limit generalizability. Biological subgroups were modest in size, reducing precision of subgroup estimates. Developmental assessment relied on parent-report methodology, which may introduce reporting variability.

Socioeconomic variables were not systematically modeled, and residual confounding by environmental context cannot be excluded. Given the established influence of early environmental factors on developmental outcomes (22, 40, 41, 42), future research incorporating structured socioeconomic indicators would provide a more comprehensive framework.

In addition, the original ASQ-3 technical documentation does not provide detailed sex-stratified normative subgroup distributions for all six-month domains, limiting the granularity of cross-cultural normative comparison.

### 4.8 Conclusion

ASQ-3 outcomes at six months are broadly consistent with cross-cultural similarity in a Bulgarian infant cohort relative to U.S. normative benchmarks. Observed deviations were small and did not indicate structural developmental divergence during early infancy.

In contrast, biological risk factors—particularly low birth weight—were associated with more substantial developmental variability, underscoring the importance of early neurobiological influences.

These findings support the continued clinical use of ASQ-3 in the Bulgarian context while emphasizing the importance of integrating perinatal risk profiling into early developmental surveillance frameworks.

## Data Availability

The raw data supporting the conclusions of this article will be made available by the authors, without undue reservation.
